# Serum Cereblon (CRBN) Levels Predict Long Term Post- Lenalidomide-Dexamethasone Survival in Multiple Myeloma (MM) Patients and Correlate with Disease Characteristics

**DOI:** 10.3390/ijms26136341

**Published:** 2025-06-30

**Authors:** Annita-Ioanna Gkioka, Alexandros Gkiokas, Mavra Papadatou-Gigante, Alexandros Alexandropoulos, Thomai-Marina Tryfou, Aspasia Koudouna, Vasiliki Bartzi, Marie-Christine Kyrtsonis

**Affiliations:** Hematology Section of First Department of Propedeutic Internal Medicine, National and Kapodistrian University of Athens, 11527 Athens, Greece; anni.iwan.gk@gmail.com (A.-I.G.); alexandergkiokas12@gmail.com (A.G.); mavra90@yahoo.com (M.P.-G.); al.alexandropoulos@gmail.com (A.A.); thommais@hotmail.com (T.-M.T.); aspakoud@hotmail.gr (A.K.); vbartzi@yahoo.com (V.B.)

**Keywords:** serum cereblon, CRBN, immunomodulatory drugs, lenalidomide, multiple myeloma, prognosis, response biomarkers

## Abstract

Serum cereblon (CRBN) has been proposed as a target protein for immunomodulatory drugs (IMiDs). IMiDs are one of the backbone treatment options in multiple myeloma (MM), rendering CRBN an intriguing candidate for use as a biomarker in clinical settings. Ninety-two (92) MM patients, mostly relapsed/refractory and a few at diagnosis, were included in the study, from lenalidomide–dexamethasone (LD) initiation until last follow-up or death. Median CRBN at LD initiation (*N* = 68) treatment was 247 pg/mL (range, 0–9760 pg/mL), at the time of best response (BR) status (*N* = 59) 142.5 pg/mL (range, 0–9940 pg/mL) and in patients with relapse/refractory MM to LD regimen (N = 54) 298 pg/mL (range, 0–9840 pg/mL). CRBN in healthy individuals was almost undetectable and significantly lower compared to the CRBN at LD initiation (*p* = 0.003), at BR to LD (*p* = 0.012) and at relapse to LD (*p* = 0.002). CRBN was significantly lower at BR in contrast to LD initiation and relapse to LD (*p* = 0.04, *p* = 0.028). High levels of CRBN at treatment initiation correlated with early relapse to LD (≤12 months) (*p* = 0.03). Seven-year survival was improved in patients with CRBN levels below median measured at the time of LD initiation (*p* = 0.013) as well as at BR (*p* = 0.032). CRBN was associated with treatment response and is predictive of survival after LD.

## 1. Introduction

Multiple myeloma (MM) is a hematological malignancy characterized by monoclonal bone marrow plasma cell infiltration and the production of paraprotein which may lead to clinical and laboratory presentation of hypercalcemia, renal failure, anemia, and bone lesions, commonly referred in daily clinical practice as CRAB criteria. Over the last 20 years, the median survival for multiple myeloma (MM) patients has improved significantly to more than seven years. The introduction in clinical practice of proteasome inhibitors (PIs) and immunomodulatory drugs (IMiDs) was fundamental for this accomplishment [1]. Likewise, the utilization of combinations of these two drugs, as well as the additive value of monoclonal antibodies such as Daratumumab, has improved patient outcomes during the previous decade. Furthermore, bispecific antibodies as well as chimeric antigen receptor (CAR) T-cell therapy are both standard approved therapies for multiple myeloma now and have shown encouraging results in clinical studies. Lenalidomide–dexamethasone (LD) combination was originally authorized for use in relapsed/refractory MM (RRMM) patients in 2006, and in newly diagnosed MM in 2015 [2]. Lenalidomide treatment in front line is widely used, and lenalidomide refractoriness at first relapse represents a challenge [1]. Even though a drug research revolution is underway, MM patients eventually relapse, indicating that myeloma cells can develop resistance to all available therapies, leading to shorter survival. The fact that relapse/refractoriness impacts following treatment options is indeed a matter of severe concern.

Cereblon (CRBN) was firstly described by Higgins et al. in 2004, as a 442 amino acid protein, of 51 kDa, having an ATP-dependent Lon protease function encoded by a locus in chromosome 3p; mutation in this gene was associated with mild mental retardation [3,4]. CRBN is expressed in many organs and represents a multifunctional protein involved in many biological processes. It is found inside the cell in the nucleus, cytoplasm, and peripheral membrane. In particular, CRBN was found to interact with large-conductance Ca++ activated potassium channel (BKCa) and chloride channel (CIC) proteins regulating cell excitability in neurons. Furthermore, its importance has been identified in cell metabolism in the regulation of 5′ adenosine monophosphate-activated protein kinase and in autoimmune diseases with the regulation of CD4 T lymphocytes [5]. A breakthrough report from Ito et al. 2010 described CRBN as the primary target of thalidomide teratogenic effects. CRBN was identified as a part of an E3 ubiquitin ligase complex involving DNA damage-binding protein-1 (DDB1), Cullin 4 (Cul4A or Cul4B), and regulator of Cullins 1 (RoC1) with proteolysis activity [6]. Inhibition of CRBN auto-ubiquitination and E3 ubiquitin ligase activity of the cereblon-containing complex leads to downregulation of fibroblast growth factor 8, which is important for limb outgrowth [6]. Further research underlying anti-MM activity of IMiDs revealed two different mechanisms, a ubiquitin-dependent pathway with many factors playing a pivotal role such as ikaros (IKZF1) and aiolos (IKZF3), interferon regulatory factor 4 (IRF4) and interleukin-2 (IL2) [7], as well as an ubiquitin-independent pathway in which IMiDs bind to CD147-MCT1 complex instead of CRBN, producing an obstacle in tumor growth [8].

Uncovering mechanisms of CRBN action in MM, researchers supported its use in clinical practice as a biomarker to IMiD treatment. In fact, studies of BM biopsies revealed an association between CRBN suppression and resistance to IMiDs. (9). Next-generation analogues, termed cereblon E3 ligase modulators (CELMoDs), iberdomide and mezigdomide, are currently being assessed in phase III clinical trials. However, limited data exist about CRBN levels in the serum of MM patients.

Herein, the aim of the present study was to analyze CRBN levels in the serum of MM patients treated with LD, its prognostic impact, and its relation with other clinical characteristics.

## 2. Results

Sera samples collected from concerned patients treated with LD from 2006–2016 with available sera at LD initiation (68 patients), at Best Response (59 patients), and during relapse/refractoriness (54 patients) to MM were included in the analysis. The median age of the patients was 70 years (range, 43–90) with 52 (56%) men and 40 (44%) women. The Ig type was IgG in 58 (64%), IgA in 20 (22%), light-chain in 10 (11%), and other in 4 (3%) patients, respectively. Twenty-nine patients (32%) were staged as ISS 1, 19 (20%) as ISS 2, and 44 (48%) as ISS 3. LD regimen was administered as induction treatment in 8 (9%) patients, second line in 36 (39%), third line in 23 (25%), fourth line in 14 (15%), and 5th– 9th line treatment in 11 (12%) patients. The median OS was 76 months (6–376), and the median TTNT was 14 months (2–110). Notably, prior exposure to IMiDs did not appear to impact CRBN levels at the initiation of LD treatment. Table 1 and Table 2 provide a summary of all the patients’ baseline characteristics and the course of the disease.

### 2.1. CRBN Levels Distribution Receiving Lenalidomide Treatment

**Figure 1:**
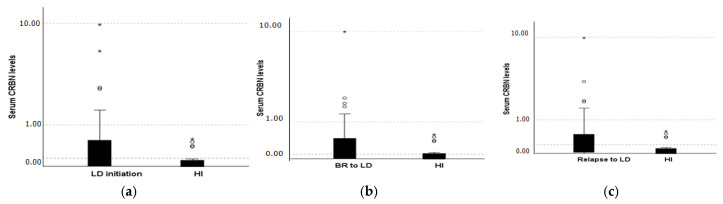
CRBN levels at (**a**) LD initiation, (**b**) best response (including responders and non-responders to LD) and (**c**) relapse to LD compared to HI (healthy individuals). (^o^) Indicates an outlier value (value outside 1.5 × interquartile range) and (*) indicates an extreme outlier (value outside 3 × interquartile range).

**Figure 2:**
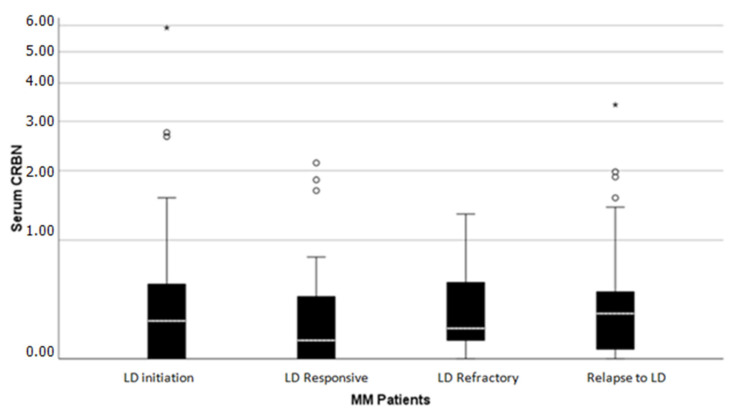
Serum CRBN levels at LD initiation, BR, and Relapse to LD. (^o^) Indicates an outlier value (value outside 1.5 × interquartile range) and (*) indicates an extreme outlier (3 × interquartile range).

Median levels of CRBN at LD initiation were 247 pg/mL (range, 0–9760 pg/mL), at the time of best response status 142.5 pg/mL (range, 0–9940 pg/mL), and in patients with relapse/refractory MM to LD regimen 298 pg/mL (range, 0–9840 pg/mL), while in normal controls CRBN levels were almost undetectable with a median value of 0 pg/mL (range: 0 to 580 pg/mL) (Figure 1). Median CRBN levels among BR patients were 193 pg/dl for LD refractory patients and 113 pg/mL for LD responsive ones. Eight out of 20 normal controls had measurable CRBN that ranged from (0–580) pg/mL. CRBN levels of healthy individuals were significantly lower compared to the serum levels of CRBN at LD initiation (*p* = 0.003), at BR (*p* = 0.012), and at Relapse to LD (*p* = 0.002) (Figure 2). Serum CRBN levels were significantly higher at LD initiation compared to CRBN levels at BR (N = 48, *p* = 0.04). No statistically significant difference (*p* = 0.479) was observed between LD initiation (median, 247 pg/mL) and Relapse to LD (median, 298 pg/mL) (*p* = 0.028) (Figure 2). LD refractory individuals had circulating CRBN levels that did not differ significantly from serum levels upon LD relapse (*p* = 0.694).

### 2.2. CRBN Correlations with Disease Characteristics

CRBN levels at LD initiation positively correlated with increased BMINF ≥ 60% (*r* = 0.268, *p* = 0.05) and abnormal LDH (*r* = −0.486, *p* = 0.001). No correlation was observed with anemia, renal failure, hypercalcemia, bone disease, plasmacytomas, thrombocytopenia, or biochemical relapse present at LD initiation. Furthermore, high CRBN serum levels at LD initiation correlated with patients experiencing an early relapse (≤12 months). Although intriguing, these patients responded to LD (≥PR) rather well (*r* = −0.258, *p* = 0.03), considering the limited time to relapse they experienced. Serum CRBN levels below median at BR correlated with better response to the next line after LD treatment (*r* = −0.456, *p* = 0.025). LDH ≥ ULN measured at BR correlated also with the serum CRBN levels at BR to LD (*r* = −0.411, *p* = 0.004). The median CRBN ratio (LD initiation/BR to LD was 1.37. A ratio of CRBN (LD initiation/BR to LD) ≥ 1.37 correlated with anemia (*r* = −0.330, *p* = 0.046) and a ratio of CRBN (LD initiation/BR to LD) ≥ 1 was associated with relapse to LD after 5 years (*r* = −0.338, *p* = 0.041). Furthermore, the CRBN ratio calculated in patients refractory to LD (0.31) predicted better the patients with response to LD ≥ PR and the duration of response with a strong correlation (*r* = −0.875, *p* ≤ 0.001) but not the relapse of patients to LD after 5 years of treatment (*r* = −0.108, *p* = 0.457). Correlations of serum CRBN with disease characteristics are presented in Table 3.

### 2.3. Survival Analysis

**Figure 3:**
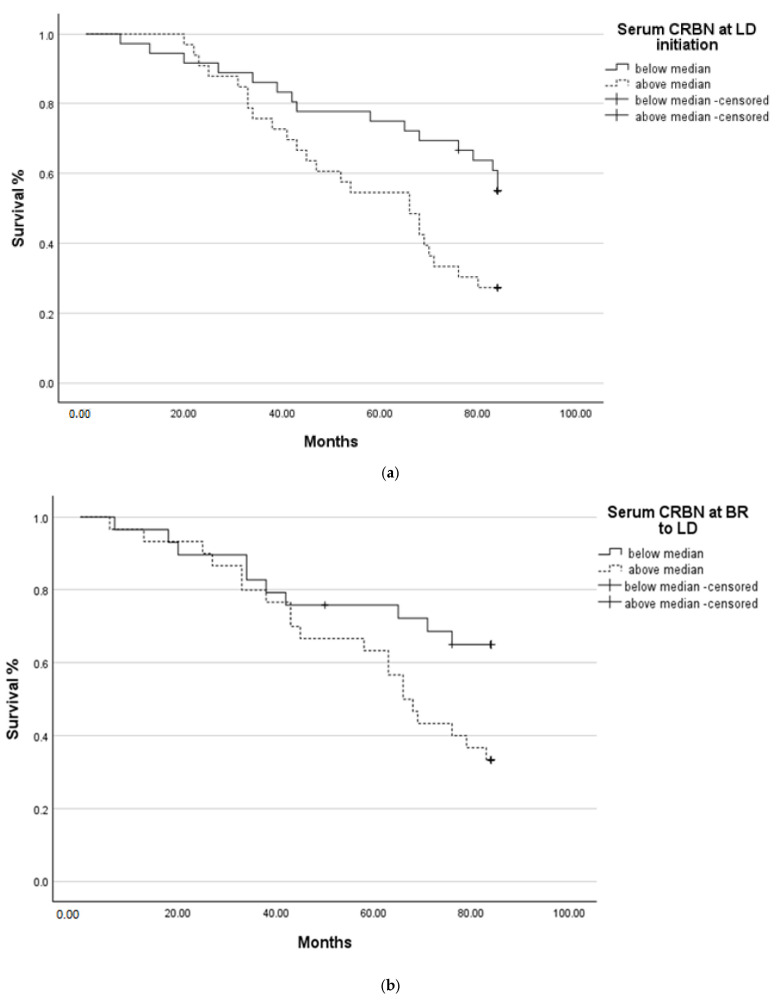

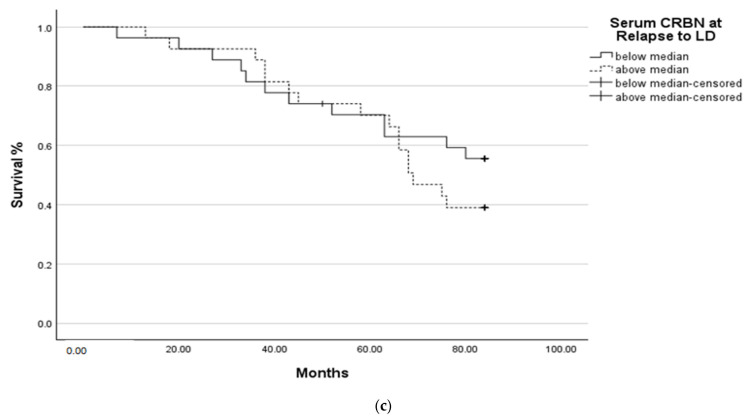
Seven-year OS based on serum CRBN levels at LD initiation (**a**), BR (**b**), and Relapse to LD (**c**). The “+” symbol indicates censored observations.

**Figure 4:**
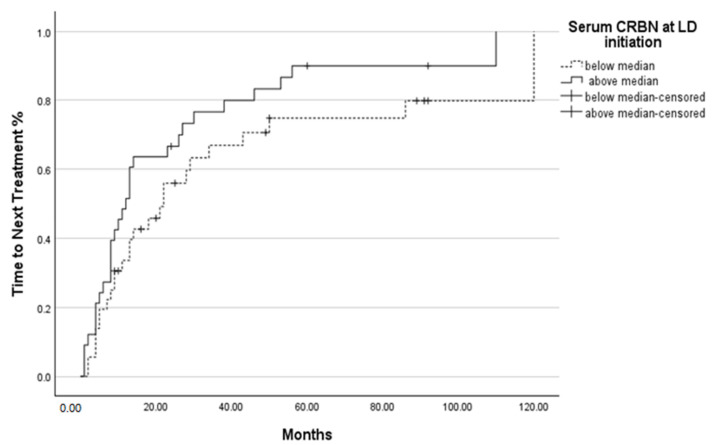
Time to Next Treatment based on CRBN levels at LD initiation. The “+” symbol indicates censored observations.

Seven-year survival was improved in patients with CRBN levels below the median at the time of LD initiation (*p* = 0.013), during the best response (*p* = 0.032) (Figure 3a,b) but not at relapse/refractory patients to LD (*p* = 0.357) (Figure 3c). The time to next treatment did not differ among CRBN levels at LD initiation (*p* = 0.121) and BR to LD (*p* = 0.074) (Figure 4).

## 3. Discussion

Lenalidomide constitutes one of the backbone agents in MM. More than a decade ago, CRBN was identified as a target protein of IMiDs [6]. Since then, other fundamental processes of CRBN regulation have been proposed, including the E3-ligase complex control, CRBN gene isoforms, and epigenetic regulation. Furthermore, a few studies have investigated the expression levels of CRBN in MM cell lines [9] and bone marrow samples [10,11,12] in order to uncover a potential new targeted treatment or the prognostic importance of this component. To our knowledge, this is the first study of CRBN levels in the serum of MM patients treated with LD. We report serum CRBN levels at LD initiation (*p* = 0.003), BR (*p* = 0.012), and relapse to LD (*p* = 0.002) to be considerably higher than in controls. Our finding that serum CRBN levels are significantly higher in MM patients compared to healthy controls suggests that malignant plasma cells may contribute to circulating CRBN. Although CRBN is primarily an intracellular protein without a classical secretion signal, its presence in serum may result from passive release due to increased tumor cell turnover or apoptosis, or from active export via non-classical mechanisms such as exosome cleavage. Further investigation, including ELISA-based analysis from MM cell lines, may help confirm the source and clarify the mechanism of CRBN secretion in the context of MM. In other reports, CRBN expression has been evaluated by real-time PCR in the bone marrow of patients prior to IMiD therapy, and it was significantly higher than in controls (*p* < 0.001) [13,14]. Importantly, bone marrow and serum CRBN measurements carry distinct biological implications: bone marrow CRBN is more indicative of tumor cell-intrinsic expression and potential IMiD sensitivity, whereas serum CRBN may reflect overall tumor burden or disease activity.

Measuring serum CRBN, we observed significant depletion of serum CRBN levels, moving from LD treatment initiation to Best Response to LD. An increase in CRBN serum levels was seen when the patient relapsed to LD. In patients retreated with LD, CRBN remained elevated. According to the limited data in the literature, reduced CRBN mRNA expression levels have been related to relapse/refractory MM [9,13,15,16]. More particularly, in MM cell lines, CRBN mRNA expression was examined after continuous exposure for 6 months to lenalidomide 1 mM, as well as in cultures treated sequentially with 1 mM for 2 months followed by 10 mM lenalidomide for 4 months. The last escalation dose of 10 mM lenalidomide was used to explore the acquisition of resistance to lenalidomide, which was greater than the one clinically reached in the plasma. In the preceding study, CRBN mRNA expression was observed to be reduced in both concentrations of lenalidomide dosage [9].

Additionally, high CRBN expression levels in the bone marrow (BM) were related to favorable response to IMiD treatment [13,15,16]. In this context, CRBN expression was studied in 46 MM patients and was associated with response to thalidomide-dexamethasone therapy. In the above investigation, levels of CRBN were decreased, shifting from complete to partial response, and patients who were not sensitive to thalidomide experienced further reduction in CRBN expression levels. This last group of patients was relatively small, with only 7 cases [14]. Similar to the above findings, another study group [10] examined CRBN expression in 49 previously untreated MM BM specimens receiving LD. The study group observed that patients with stable disease or progressive disease had significantly lower expression than the patients who were responsive to therapy. Bila et al. 2018 [13] examined CRBN gene expression in the BM of 77 patients treated with thalidomide-based regimens and 15 with bortezomib regimens. In the patients with a treatment response to thalidomide, a significant correlation was observed with high CRBN expression, mainly in the group treated with thalidomide-based combinations, while it was not observed in the bortezomib group. Of note, from this cohort, only 3 patients were in PD. Although the above studies suggest that CRBN expression is lowered, the sample size of patients with stable or progressing disease was rather modest.

Lower gene expression of the CRBN was also observed in 55 relapsed/refractory MM patients treated with Pomalidomide-Dexamethasone [10].

According to IHC studies, Franseen et al. 2018 [11] observed a substantial decrease in CRBN BM expression of 55 MM patients, who became resistant to lenalidomide. Notably, 23% of 55 patients who developed resistance to lenalidomide did not have CRBN expression alteration, and half of them were primary refractory to lenalidomide-based treatment, compared to only 13% in patients with a decrease in CRBN expression, while there was no difference in baseline CRBN expression between primary refractory patients and patients with an initial response to lenalidomide-based treatment. Other explanations for lenalidomide resistance are most likely involved in these individuals. In another attempt by Huang et al. to investigate CRBN expression in MM, it was shown that the IHC CRBN (+)-positive stain was associated with a response to LD and thalidomide-dexamethasone treatment. From this cohort study, there was a subset of patients (21% relapsed/refractory MM and 25% newly diagnosed MM, respectively) who did not respond to the LD and TD regimen, despite expression of CRBN protein within the myeloma cells, suggesting the possibility of mechanisms of resistance that do not involve CRBN [16]. We showed that patients with a noteworthy response (≥PR) and higher serum CRBN levels experience early relapse to LD (*p* = 0.032). MM patients who relapse early are of growing concern since survival outcomes remain poor, and moreover the depth of response to therapy [12] implies that prolonged survival is related to other factors in addition to the extent of response to treatment.

In this regard, another resistance mechanism to lenalidomide is the activation of the Wnt/β-catenin pathway by lenalidomide treatment [17], which affects downstream targets, such as CCND1 and MYC. Furthermore, substrate receptors that bind DDB1-CUL4 apart from CRBN exist and compete for binding to DDB1. Thus, these receptors may be involved in different cell functions including resistance, but these functions need further investigation [18]. Other rare mechanisms of lenalidomide resistance may include point mutations and acquired deletion of the CRBN gene. Missense mutations in CRBN resulted in varying levels of function, including total loss for all IMiDs, no effect on CRBN function, and agent-dependent effect. In fact, these mutations may be present at the time of resistance to IMiDs [19]. This suggests that the low levels of CRBN gene expression might be explained by the fact that the PCR-based methods fail to identify these mutant alleles.

In the present study, levels of CRBN above the median correlated with adverse prognosis in MM patients treated with LD, and though unforeseen, maybe it is explained by mechanisms of protein release in the serum. We were studying the protein that was set from the cells and circulates freely in the blood. The same has been observed for other soluble factors, syndecan-1 protein, for instance, the high expression of which correlates with better prognosis than the low expression [20]. On the contrary, high soluble levels of syndecan-1 confer poor prognosis [21]. Indeed, proteins follow several ways of shedding via classical secretory pathways and other unconventional pathways (protein cleavage) or through exocytosis [22]. Despite the fact that CRBN has been found in the nucleus, cytoplasm, and peripheral membrane of numerous tissue cells, the specific method of shedding from the cell has yet to be determined. In fact, in resistant MM cell lines, extracellular vesicle production and adhesion abilities were significantly higher than in lenalidomide-sensitive MM cells, as demonstrated by Yamamoto et al. [23]. Also, alternative splicing of CRBN mRNA in MM produces several isoforms, some of which may not even be translated [24,25]. Further, cases of high cereblon expression in patients with acquired resistance to IMiDs have been documented, upholding the hypothesis of resistance to the drugs with a more than one-step event; in this case, downregulation of CRBN might represent a secondary ‘passenger’ phenomenon [26].

Higher CRBN serum levels were associated with BMINF (≥60%) (*r* = 0.265, *p* = 0.05). Serum CRBN levels were likewise raised in our patients with high LDH (*r* = 0.486, *p* = 0.001), a marker of high tumor burden and aggressive disease. While LDH serves as a general prognostic biomarker in MM, serum CRBN may provide an additional, therapy-specific insight, particularly in the context of IMiD-based treatment. As the molecular target of IMiDs, CRBN is mechanistically linked to treatment response, unlike LDH. Thus, serum CRBN may reflect both disease activity and sensitivity to IMiDs, offering complementary prognostic value. From available data in bone marrow samples, CRBN expression reversely correlated with (ISS) [9,14], serum beta-2-microglobulin (B2M), and serum albumin [14], while another study observed that increased levels of CRBN expression were associated with higher B2M (*r* = 0.66, *p* = 0.001) of newly diagnosed MM patients [15]. In addition, our analysis revealed that a higher CRBN serum ratio between LD initiation and BR (≥1.37) was significantly associated with anemia and earlier relapse to LD, suggesting that an increase in circulating CRBN may reflect disease progression or tumor stress. Interestingly, while a ratio ≥1 was weakly associated with relapse after 5 years, using as a cutoff the median value of CRBN LD initiation/BR ratio calculated only from patients refractory to LD (median CRBN LD initiation/BR ratio; 0.31) showed a strong correlation for response (≥PR) and relapse within 12 months (*r* = −0.875, *p* < 0.001), indicating its potential as a dynamic biomarker for treatment outcomes.

In the context of survival outcomes, there are contradictory findings regarding the prognostic value of CRBN expression. Despite reports indicating significant correlation between high CRBN expression levels in BM and increased PFS [10,13,27], this was not validated in OS newly diagnosed or relapsed/refractory MM patients undergoing IMiDS, with some researchers confirming the association and others reporting no association [18,27,28]. In addition, the impact of CRBN expression to OS was eliminated when ISS and cytogenetics were taken into account [28,29]. In a recent study, patients with high CRBN expression in BM receiving IMiD treatment-based therapy showed improved PFS and OS compared to patients receiving proteasome inhibitor-based therapy [29]. This indicates that CRBN expression is specifically relevant to the mechanism of action of IMiDs, which rely on CRBN to exert their anti-myeloma effects. IMiDs bind to CRBN and redirect its E3 ubiquitin ligase activity to degrade key transcription factors (e.g., IKZF1/3), leading to myeloma cell death. Therefore, high CRBN expression enhances IMiD efficacy, while its level is not functionally significant in patients receiving non-CRBN-targeting drugs like PIs. The results of the aforementioned studies are summarized in Table 4.

We observed that seven-year survival following LD treatment was improved in patients with CRBN levels below median at the time of LD initiation (*p* = 0.013) and during best response (*p* = 0.032) (Figure 4). Although TTNT was, in fact, rather valuable over 14 months (range, 2–110) in our research, CRBN levels did not differ substantially across patients.

While our findings suggest that serum CRBN levels at LD initiation and at BR may help predict response duration and guide treatment decisions, several limitations must be acknowledged. Most patients today receive lenalidomide in combination with proteasome inhibitors or monoclonal antibodies, both in frontline and relapsed settings. It remains unclear whether the prognostic value of serum CRBN is preserved in these combination regimens, or if such combinations may overcome the negative impact of high CRBN levels. Additionally, further research is needed to determine whether CRBN levels at relapse predict response to subsequent therapies, such as pomalidomide. These aspects warrant prospective validation in larger, treatment-diverse cohorts.

## 4. Patients and Methods

A total of 92 newly diagnosed and relapsed/refractory MM patients in our unit were included in the study. The patients were enrolled from LD initiation until last follow-up or death. Medical records were reviewed after patients’ informed consent was obtained. Clinical characteristics and laboratory tests such as hemoglobulin (Hb), creatinine (Cr), calcium (Ca), paraprotein levels (Ig), free light chain levels (FLCs), serum-free light chain ratio (FLCR), lactate dehydrogenase (LDH), bone marrow infiltration (BMINF), B2-microglobulin (B2M), and albumin (alb) were collected and stored for statistical analysis. All patients were staged according to the international staging system (ISS) [30], and clinical response evaluation was based on the international myeloma working group (IMWG) criteria [31,32]. Patients’ sera were drawn, kept frozen, and retrospectively analyzed. Twenty healthy individuals’ serum samples were also tested. CRBN was measured at different time points. More particularly, CRBN was measured at (1) initiation of LD treatment (68 patients), (2) best response evaluation, including responders and non-responders to LD (59 patients), and (3) relapse/refractory to LD regimen (54 patients). All patients were evaluated with blood smear and lab tests, during the above time points. Fifty-four patients with data available at all time points were further evaluated by statistical analysis between these three time periods. Fifteen individuals were refractory to LD, whereas forty-four patients with measurable CRBN at BR responded to therapy. To determine the effect of the shift in CRBN serum levels from LD initiation to BR, we calculated the ratio (CRBN LD initiation/BR to LD). The median of CRBN serum levels at each time point (LD initiation, BR, and relapse to LD) was used as a cutoff point for the survival analysis.

### 4.1. Elisa Analysis

CRBN was measured by commercially available ELISA kits (Cloud-Clone Corp), according to the manufacturer’s instructions. In short, the serum of the patients and HI as well as the standards provided by the manufacturer, were added to the appropriate wells of a pre-coated 96-cell microplate with an antibody specific to CRBN. Next, avidin conjugated to horseradish peroxidase (HRP) was added to each microplate well and incubated. After TMB substrate solution was added, an enzyme-substrate reaction resulted in a change in color of the wells that contained CRBN, biotin-conjugated antibody, and enzyme-conjugated avidin. The reaction was terminated by the addition of sulphuric acid solution, and the color change was measured spectophotometrically at a wavelength of 450 nm ± 10 nm. Plot software was used to construct a log–log graph (standard curve) with CRBN concentration on the y-axis and absorbance on the x-axis. The concentration of CRBN in the samples was then determined by comparing the O.D. of the samples to the standard curve. The number zero (0) was arbitrarily used for the presentation of undetectable values.

### 4.2. Statistical Analysis

Descriptive statistics were applied for continuous variables, and categorical variables were displayed as frequencies. Nonparametric variables were compared by the Mann–Whitney U test. Correlations between clinical parameters of MM patients and CRBN were calculated using the Spearman (r) correlation method. Kaplan–Meier curves were plotted for overall survival (OS) and time to next treatment (TTNT) according to CRBN levels of patients and then compared by the log-rank test. The median was selected as a cutoff point for the statistical analysis. All the aforementioned statistical tests were performed with the use of SPSS v28.0 software, and values lower than 0.05 were considered statistically significant.

## 5. Conclusions

In conclusion, we showed that serum CRBN, as the target protein of IMiDs, was associated with disease features, survival in MM patients treated with Lenalidomide, and responsiveness to therapy, making this protein crucial for MM patients receiving Lenalidomide. Understanding the mechanism underlying this protein fluctuation in response to IMiD therapy may pave the way for future treatment decisions.

## Figures and Tables

**Table 1:** MM patients’ characteristics at LD initiation.

Variable	Patients N = 92	Percentage (%)
Age	70 (range, 43–90)	
Sex		
M	52	56%
F	40	44%
Ig type		
IgG	58	64%
IgA	20	22%
Light-Chain	10	11%
Biclonal Ig	3	2%
IgD	1	1%
International Staging System		
ISS 1	29	32%
ISS 2	19	20%
ISS 3	44	48%
Clinical presentation		
Hypercalcemia	3	3.2%
Renal Failure (Cr ≥ 2 g/dL)	15	16.3%
Anemia (Hb ≤ 10 g/dL)	35	38%
Bone Lesions (≥1)	39	42.4%
Paraosseous Plasmacytomas (≥1)	3	3.2%
Thrombocytopenia	8	8.7%
LDH ≥ ULN	12	13%
BMINF ≥ 60%	37	40.2%
RRMM with biochemical relapse	22	23.9%
Treatment line		
1st	8	9%
2nd	36	39%
3rd	23	25%
4th	14	15%
5th–9th	11	12%

Ig: immunoglobulin, ISS: international staging system, LDH: lactate dehydrogenase, ULN: upper limit number, BMINF: bone marrow infiltration, Cr: creatinine, Hb: hemoglobin, RRMM: relapsed/refractory MM patients.

**Table 2:** Patients’ MM course.

Variable	Patients N = 92 (Range)	Percentage (%)
**Treatment Lines Prior to LD** IMiD exposed • MPT • TD • Thalidomide Maintenance • LD		
	37	40.2%
	7	7.5%
	22	24%
	8	8.7%
	4	4.4%
IMiD naïve	51	55.4%
**Treatment Line After LD** • PI base • Conventional Chemotherapy • Salvage Treatment • IMiD based • IMiD and PI		
	30	56%
	10	19%
	2	4%
	2	3%
	10	18%
**Overall Survival**	76 months (range, 6–376)	
**Time To** **Next** **treatment**	14 months (range, 2–110)	
Patients relapsed to LD	N = 54	
• Response to LD ≥ PR	44	72.2%
• Refractory to LD	15	27.8%
**Response to** **LD**	**N = 59**	
sCR	9	17%
CR	9	17%
VgPR	11	20%
PR	10	18%
PD	15	28%
**Response to Next Treatment after LD**	**N = 34**	
Scr	2	5%
CR	5	13%
VgPR	1	2%
PR	12	35%
MR	9	26%
PD	7	20%

PI: proteasome inhibitor, IMiDs: immunomodulatory Drugs, MPT: melphalan-prednisone-thalidomide, TD: thalidomide-dexamethasone, sCR: stringent complete response, CR: complete response, VgPR: very good partial response, PR: partial response, MR: minimal response, PD: progressive disease.

**Table 3:** Correlations of serum CRBN with disease characteristics.

• Serum CRBN at LD Initiation	Correlation Coefficient (r)	*p*-Value
BMINF ≥ 60%	0.265	0.05
LDH ≥ ULN	0.486	0.001
Response	0.074	0.580
Response ≥ PR to LD	0.160	0.169
Relapse ≤ 12 months	0.097	0.414
Response ≥ PR to LD and Relapse ≤ 12 months	−0. 258	0.032
• **Serum CRBN at BR to LD**		
Response to Next Treatment after LD	−0.456	0.025
LDH ≥ ULN	0.411	0.004
• **Serum CRBN at Relapse to LD**		
Anemia (Hb ≤ 10 g/dL)	−0.330	0.046
Response to Next Treatment after LD	−0.344	0.063
• **Serum CRBN (LD initiation/BR to LD ratio ≥ 1.37)**		
Anemia (Hb ≤ 10 g/dL)	−0.330	0.046
• **Serum CRBN (LD initiation/BR to LD ratio ≥ 1)**		
Relapse to LD after 5 years	−0.338	0.041
• **Serum CRBN (LD initiation/BR to LD) ratio ≥ 0.31)**		
Response ≥ PR to LD and Relapse ≤ 12 months	−0.875	<0.001
Relapse to LD after 5 years	−0.108	0.457

BMINF: Bone Marrow Infiltration, LDH: lactate dehydrogenase, ULN: upper limit number, Serum CRBN (LD initiation/BR to LD) ratio ≥ 0.31): this value corresponds to the median value of serum CRBN ratio calculated in patients refractory to LD.

**Table 4:** Prognostic significance of CRBN from different studies.

*N*	Author	Patients/Status	Method	Regimen	FU Duration	Response Predicted	PFS	OS
**1**	Bila et al. 2016 [13]	77/NDMM	RT-PCR	thalidomide combinations	27 months (range, 4–42 months)	Decreased CRBN—poor prognosis (*p* = 0.028)	*p* = 0.017	NS
**2**	Heintel et al. 2013 [15]	49/NDMM	RT-PCR	LD	4 (range: 3–37) cycles	Decreased CRBN–poor prognosis (*r* = 0.48), (*p* < 0.001)	NS	NS
**3**	Huang et al. 2014 [16]	85/NDMM and RRMM	IHC	LD and TD	LD—28 months TD –not reported	CRBN negative stain-poor prognosis for LD (*p* = 0.005) and TD (*p* = 0.005)	LD NS TD NS	LD NS TD NS
**4**	Schuster et al. 2014 [10]	55/RRMM	GEP	pomalidomide-dexamethasone	PFS—3.0 vs. 8.9 months OS −9.1 vs. 27.2 months	No relation	*p* = 0.0006 (lowest quantile)	*p* = 0.01 (lowest quantile)
**5**	Klimowitz et al. 2012 [27]	42/NDMM and RRMM	IHC	LD	22.4 months (range 0.72–65.6)	NT	*p* = 0.012	*p* = 0.044
**6**	Broyl et al. 2013 [28]	96/NDMM	GEP	HOVON65/GMMGHD4 trial. Thalidomide maintenance	24 months	NT	*p* = 0.005	*p* = 0.04
**7**	Lee BH et al. 2021 [29]	130/NDMM	IHC	IMiD	PFS-29 months OS-NR	NT	*p* = 0.03	*p* = 0.01

NS: not significant, NT: Not tested, NR: Not Reached, IHC: Immunohistochemistry, GEP: Gene Expression Profile, RT-PCR: Real-Time Polymerase Chain Reaction, TD: thalidomide-dexamethasone, NDMM: Newly diagnosed Multiple Myeloma, RRMM: Relapsed/Refractory Multiple Myeloma.

## Data Availability

Data are contained within the article.
